# Effects of hormone replacement therapy on glucose and lipid metabolism in peri- and postmenopausal women with a history of menstrual disorders

**DOI:** 10.1186/s12902-021-00784-9

**Published:** 2021-06-15

**Authors:** Saisai Li, Linjuan Ma, Yang Song, Jiehong Zheng, Yuqun Cai, Hong Xu, Peiqiong Chen, Wenxian Xu, Yizhou Huang, Tongyun Qi, Chunming Li, Ketan Chu, Yibing Lan, Ling Xu, Jianhong Zhou

**Affiliations:** 1 Department of Gynecology, Women’s Hospital, Zhejiang University School of Medicine, 1st Xueshi Road, Hangzhou, 310006 China; 2 Maternal and Child Health Hospital, Jiangbei District, Ningbo, China; 3 Maternal and Child Health & Family Planning Service Center, Gongshu District, Hangzhou, China; 4 Maternal and Child Health Hospital, Tongxiang, Zhejiang China; 5 Zhejiang Maternal, Child and Reproductive Health Center, 256 Wantang Road, Hangzhou, 310012 China

**Keywords:** Hormone replacement therapy, Glucose and lipid metabolism, Menstrual disorders, Peri- and postmenopausal women

## Abstract

**Background:**

Previous studies have indicated that women with a history of menstrual disorders have an increased risk of metabolic and cardiovascular diseases. This has been attributed to the high proportion of polycystic ovary syndrome (PCOS) among this group. The favorable effects of hormone replacement therapy (HRT) on serum lipid profiles and glucose homeostasis in postmenopausal women is widely accepted. Whether HRT can also show positive effects on metabolic homeostasis in menopausal women with prior menstrual disorders (a putative PCOS phenotype) has not been reported yet. The aim of the study was to compare the effects of HRT on glucose and lipid metabolism in peri- and postmenopausal women with prior menstrual disorders and controls who did not have prior menstrual disorders.

**Methods:**

A retrospective multicenter study was conducted including 595 peri- and postmenopausal women who received HRT at four hospitals in the Zhejiang Province from May 31, 2010 to March 8, 2021. Participants were divided into the Normal menstruation group and the Menstrual disorders group according to their prior usual menstrual cycle pattern. Glucose and lipid metabolism indicators were assessed at baseline and after HRT. The results were compared between and within the groups, and data from peri- and postmenopausal women were analyzed separately.

**Results:**

HRT significantly decreased fasting insulin and homeostasis model assessment of insulin resistance in perimenopausal users, and fasting plasma glucose levels in postmenopausal users with prior menstrual disorders, compared with baseline. Furthermore, HRT decreased low-density lipoprotein cholesterol, total cholesterol, fasting insulin, fasting plasma glucose and homeostasis model assessment of insulin resistance in both peri- and postmenopausal controls, compared with baseline. Nevertheless, no significant differences were observed in any of the glucose or lipid metabolism indicators at baseline and follow-up, as well as changes from baseline levels between menopausal women with and without prior menstrual disorders.

**Conclusions:**

HRT shows more obvious within-group improvements in glucose and lipid metabolism in controls, but there is no significant between-group difference. Further prospective studies are required for confirmation.

## Introduction

Menstrual irregularity is frequently considered a sign of ovulatory dysfunction and underlying insulin resistance [1–3]. Oligomenorrhea is correlated with hyperinsulinemia and increased risk of diabetes mellitus [2, 4, 5]. These phenomena may reflect polycystic ovary syndrome (PCOS), a frequent cause of oligomenorrhea [6, 7]. The syndrome frequently affects young women but can affect women of all ages. According to the Rotterdam criteria, the diagnostic criteria of PCOS are polycystic ovaries (≥12 follicles (2–9 mm) in at least one ovary, and/or increased ovarian volume > 10 ml), anovulation, and clinical or biochemical hyperandrogenism [8]. PCOS have increased rates of multiple metabolic disturbance, such as dyslipidemia, impaired glucose tolerance, insulin resistance, and diabetes mellitus [9–11]. As the classic phenotype of PCOS improves with aging, identifying menopausal women with PCOS becomes difficult [12, 13]. Thus far, a specific phenotype for PCOS in women beyond reproductive age is not fully understood [13].

Menopause is characterized by a wide array of clinical symptoms, which are attributed to the decline and ultimate cessation of ovarian function [14]. Previous study has shown that estrogens play cardiovascular-protective roles in the premenopausal woman, their decline during the menopausal transition leads to an increased prevalence of cardiovascular disease (CVD) [15]. The alterations of the lipid profiles during perimenopause and after menopause is a major constituent contributing to CVD [16].

Up to now, extensive studies consistently demonstrated that hormone replacement therapy (HRT) was an effective method to relieve climacteric symptoms and several long-term complications due to low estrogen, such as osteoporosis, dyslipidemia, and cardiovascular disease [17–25]. However, whether the use of HRT can also show favorable effects on metabolic homeostasis in peri- and postmenopausal women with a history of menstrual disorders (a putative PCOS phenotype) has not been reported yet. Therefore, the purpose of this research was to compare the effects of HRT use on glucose and lipid metabolism in peri- and postmenopausal women with a history of menstrual disorders and controls who did not have prior menstrual disorders.

## Methods

### Study subjects and design

This was a multicenter retrospective study conducted in the gynecologic endocrinology clinics at four hospitals in Zhejiang Province from May 31, 2010 to March 8, 2021. These centers included Women’s Hospital, Zhejiang University School of Medicine, Hangzhou; Maternal and Child Health & Family Planning Service Center, Gongshu District, Hangzhou; Maternal and Child Health Hospital, Tongxiang, Zhejiang; Maternal and Child Health Hospital, Jiangbei District, Ningbo. Women seeking treatment for climacteric symptoms were asked to complete structured questionnaires to obtain data including sociodemographic characteristics, menstrual and reproductive history, disease and medication history, as well as surgery therapy history if any. Simultaneously, they received a thorough clinical evaluation, including physical and gynecological examinations, transvaginal ultrasonography, breast ultrasound or mammography and laboratory evaluations. All participants must have had an indication for HRT and received HRT for 6–12 months. The assessments were carried out at baseline and after therapy. For women with multiple follow-ups during this period, the last follow-up data were chosen to maximise the follow-up time. As different HRT regimens can affect results, we only included women taking oral estrogen. Participants were excluded if they reported (i) pregnancy or lactation, (ii) having contraindication for HRT, (iii) a history of using estrogen and progestogen, (iv) current or past history of malignancies, (v) a history of chemotherapy or radiotherapy, (vi) diagnosis of any severe systematic or major organ diseases (vii) a history of other endocrine diseases such as premature ovarian insufficiency, hyperprolactinemia, thyroid dysfunction that may cause menstrual disorders, (viii) psychological disorders and psychiatric illness (schizophrenia, depression), (ix) chromosomal diseases such as Turner syndrome, (x) a history of autoimmune disorders, (xi) allergy to any ingredients of the HRT, (xii) had missing or incomplete data at baseline and after treatment, (xiii) received other HRT regimens besides oral estrogen. Ethical approval was obtained from the Ethics Committee of Women’s Hospital, Zhejiang University School of Medicine. All participating women provided written informed consent for this study.

Among all participants, the usual menstrual cycle regularity in their reproductive years and current menstrual conditions were collected in the baseline interview. Participating women were divided into four categories in accordance with the prior usual menstrual cycle pattern as follows: normal cycle (usual cycle length of 28 ± 7d), polymenorrhea (usual cycle length less than 21 d), oligomenorrhea (usual cycle length greater than 35 d), and irregular. Women with a history of menstrual disorders in the reproductive years (oligomenorrhea and irregular cycle) were selected as cases; and women with normal menstrual cycles in the prior years were recruited as controls for this study. According to the current menstrual changes, women were considered as postmenopausal if they have experienced either natural menopause (at least 12 months since the last menstrual period) or surgical menopause (bilateral oophorectomy before natural menopause). Women were classified as perimenopausal at baseline if they reported less regular menses in the past year.

**Fig. 1:**
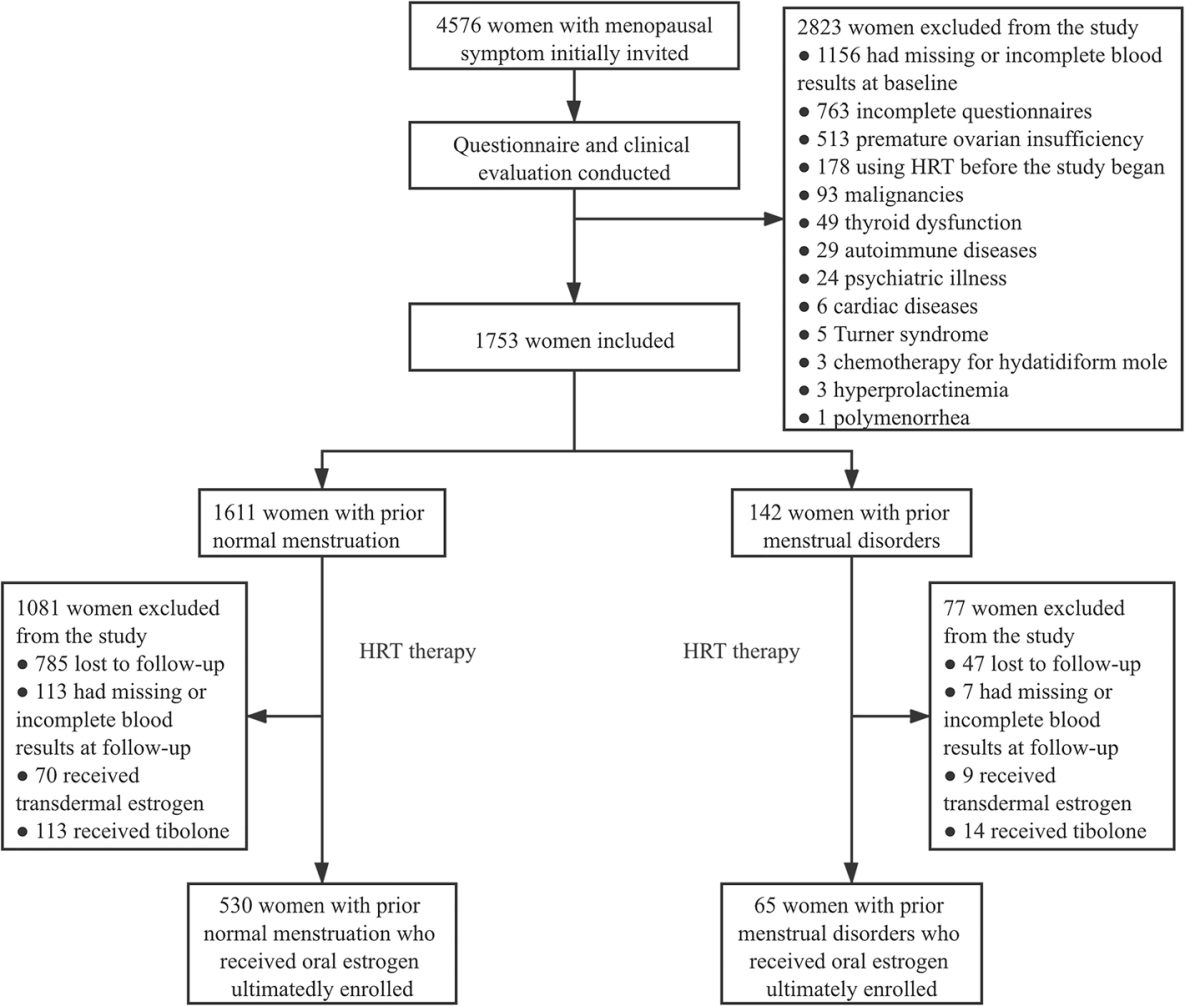
Study flow diagram. *HRT* Hormone replacement therapy

A total of 4576 women with menopausal symptoms were initially recruited from four clinical sites. After screening, 2823 were excluded according to the exclusion criteria, then 1753 were included in the present study. At 6–12 months of HRT treatment, 1158 women were withdrawn due to missing or incomplete laboratory results, lost to follow-up, receive transdermal estrogen or tibolone. Thus, 595 participants who received oral estrogen were ultimately enrolled in the present study, including 530 women with prior normal menstrual cycle, 65 women with a history of menstrual disorders (49 for oligomenorrhea and 16 for irregular menstrual cycles). The flowchart was presented in Fig. 1.

### Anthropometric indices

Each participant enrolled was measured for anthropometric data which included height, weight, blood pressure, and circumferences of the waist and hip during the baseline physical examination. According to the Chinese criteria for adults, body mass index (BMI) (calculated by the formula: BMI = weight (kg)/height(m)^2^) was defined as underweight (BMI < 18.5 kg/m^2^), normal weight (BMI 18.5–23.9 kg/m^2^), overweight (BMI 24.0–27.9 kg/m^2^), and obesity (BMI ≥28 kg/m^2^), respectively [26].

### Laboratory analyses

All women provided peripheral blood to measure glucose and lipid metabolism parameters, reproductive endocrine hormones before and after HRT treatment. Before blood sampling, participants were fasted for at least 8 h. The indexes evaluated in this paper included serum levels of fasting insulin (FIN), fasting plasma glucose (FPG), high-density lipoprotein cholesterol (HDL-C), total cholesterol (TC), low-density lipoprotein cholesterol (LDL-C), triglycerides (TG), estradiol (E2), luteinizing hormone (LH), and follicle-stimulating hormone (FSH). All these indicators were determined by a Roche Modular Analytics E170 fully automated analyzer (Roche Diagnostics, Mannheim, Germany). In addition, homeostasis model assessment of insulin resistance (HOMA-IR) (calculated by the formula: HOMA-IR = FPG*FINS/22.5) was used to evaluate insulin resistance [27].

### Statistical analysis

All statistical analyses were conducted by Statistics Package for Social Sciences 26.0 software (SPSS Inc., Chicago, IL, USA). Data are reported as mean ± standard deviation (SD), or medians (25th percentile, 75th percentiles) for continuous variables and numbers (frequencies) for categorical variables, respectively. The Kolmogorov-Smirnov test was utilized to analyze the normal distribution of continuous variables. For normally distributed data, inter-group comparisons were determined using the independent samples t-test, and intra-group comparisons were evaluated by the paired-samples t test. For non-normal distributed data, Wilcoxon signed-rank test was performed to demonstrate significant differences within groups, and Mann-Whitney U test was chosen for comparison between independent groups. Additionally, categorical variables were compared by the Chi-square test or Fisher’s exact test, as appropriate. Results with *P* < 0.05 were considered significant.

## Results

### The baseline characteristics of the participants

The present study ultimately included 595 peri- and postmenopausal women, including 530 women with prior normal menses and 65 women with a history of menstrual disorders. No significant differences in baseline characteristics were observed between those with and without a history of menstrual disorders (Table 1).

**Table 1:** Baseline clinical characteristics of women with a history of menstrual disorders and controls

Variable	Normal menstruation (*N* = 530)	Menstrual disorders (*N* = 65)	*P* value^a^
Age (years)	47.70 ± 3.94	48.38 ± 4.06	0.22
Residence			
Urban	386 (72.83%)	50 (76.92%)	0.28
Suburb	104 (19.62%)	8 (12.31%)	
Rural	40 (7.55%)	7 (10.77%)	
Menstrual status			
Perimenopause, n (%)	410 (77.36%)	46 (70.77%)	0.24
Postmenopause, n (%)	120 (22.64%)	19 (29.23%)	
Age at menarche (years)	14.51 ± 1.56	14.97 ± 1.84	0.12
Gravidity	2.81 ± 1.35	2.83 ± 1.51	0.83
Parity	1.10 ± 0.44	1.14 ± 0.50	0.74
Height (m)	1.60 ± 0.05	1.59 ± 0.05	0.28
Weight (kg)	56.02 ± 6.93	55.75 ± 6.66	0.94
BMI (kg/m^2^)	21.94 ± 2.37	22.06 ± 2.37	0.59
Underweight	22 (4.15%)	5 (7.69%)	0.27
Normal weight	404 (76.23%)	48 (73.85%)	
Overweight	97 (18.30%)	10 (15.38%)	
Obesity	7 (1.32%)	2 (3.08%)	
Waist circumference (cm)	75.98 ± 6.61	76.91 ± 7.15	0.38
Hip circumference (cm)	92.48 ± 5.05	92.27 ± 5.85	0.49
Waist to hip ratio	0.82 ± 0.05	0.83 ± 0.06	0.05
Hypertension, n (%)			
No	502 (94.72%)	60 (92.31%)	0.39
Yes	28 (5.28%)	5 (7.69%)	

Data are expressed as numbers (frequencies) or mean ± standard deviation as appropriate. *P* < 0.05 was considered significant. ^a^Comparison between groups by Mann-Whitney U test/independent samples t-test for continuous variables or by χ^2^ test/Fisher’s exact test for categorical variables

Others: *BMI* body mass index

### Reproductive endocrine indicators at baseline and follow-up

No significant differences were observed in the baseline serum levels of E2, FSH or LH between those with and without menstrual disorders in either perimenopausal or postmenopausal groups (Table 2). After HRT treatment, both perimenopausal groups presented a marked increase in serum E2 level and a significant decline in serum FSH and LH levels compared with the baseline. Nevertheless, no significant differences were noted in these indicators at follow-up or the changes from baseline levels between the two perimenopausal groups. In postmenopausal women, E2 levels increased significantly and FSH decreased markedly in both groups compared with baseline. Women with normal cycles showed a significant decrease in serum LH level at follow-up, while mean LH levels in women with prior menstrual disorders did not change significantly compared with baseline levels. Furthermore, no statistically significant between-group difference was noted after HRT therapy with regard to E2, FSH, LH or the changes from baseline levels.

**Table 2:** Reproductive endocrine parameters in women with prior menstrual disorders and controls before and after HRT

Variable	Perimenopausal			Postmenopausal		
	Normal menstruation (*N* = 410)	Menstrual disorders (*N* = 46)	*P* value^a^	Normal menstruation (*N* = 120)	Menstrual disorders (*N* = 19)	*P* value^a^
**E2 (pmol/L)**						
Before HRT	63 (28.46, 139.18)	43.52 (18.35, 110.13)	0.11	43.31 (18.35, 76.81)	38.24 (18.40, 57.49)	0.45
After HRT	195.95 (121.43, 309.67)	205.50 (129.50,440.70)	0.24	166.60 (90.67, 248.89)	98.92 (45.80, 153.00)	0.05
*P* value^b^	0.000	0.000		0.000	0.02	
Δ E2^†^ (pmol/L)	118.89 (11.23, 228.34)	164.63 (38.43, 361.75)	0.12	89.13 (27.49, 176.74)	55.61 (0.00, 112.65)	0.19
**FSH (IU/L)**						
Before HRT	69.50 (51.81, 89.60)	69.30 (55.22, 98.60)	0.37	76.73 ± 28.85	74.22 ± 35.02	0.73
After HRT	43.00 (27.68, 62.46)	43.64 (23.87, 58.39)	0.82	53.86 ± 27.01	54.93 ± 25.76	0.87
*P* value^b^	0.000	0.000		0.000	0.005	
Δ FSH^†^ (IU/L)	−23.51(−43.71, −5.51)	−28.00(−45.20, −16.01)	0.19	−22.87 ± 25.45	−19.30 ± 25.95	0.57
**LH (IU/L)**						
Before HRT	37.74 (28.80, 48.57)	39.17 (29.32, 50.67)	0.54	35.19 (27.99, 45.96)	35.03 (24.38, 43.38)	0.76
After HRT	29.04 (18.48, 39.66)	30.27 (18.74, 41.85)	0.55	30.59 (20.89, 39.74)	31.90 (17.73, 40.77)	0.94
*P* value^b^	0.000	0.001		0.000	0.09	
Δ LH^†^ (IU/L)	−9.09 ± 17.11	−8.61 ± 17.05	0.86	−6.86(−12.85, 2.81)	−2.61(−9.73, 4.50)	0.46

Data are expressed as medians (25th percentile, 75th percentiles) or mean ± standard deviation as appropriate. *P* < 0.05 was considered significant.^†^Value of the difference between the baseline and follow-up reproductive endocrine parameters (E2, FSH, and LH). ^a^*P* values are for between-group differences by Mann-Whitney U test or independent samples t-test. ^b^*P* values are for within-group differences by Wilcoxon signed-rank test or paired-samples t test

Others: *E2* estradiol, *FSH* follicle-stimulating hormone, *HRT* hormone replacement therapy, *LH* luteinizing hormone

### Lipid metabolism parameters before and after HRT therapy

No statistically significant difference was observed between women with and without a history of menstrual disorders with regard to baseline serum levels of TG, HDL-C, TC, or LDL-C in either the peri- or postmenopausal women (Table 3). At follow-up, perimenopausal women with prior normal menses showed a marked decline in serum TC and LDL-C levels, whereas HDL-C and TG levels increased significantly compared with baseline. In perimenopausal women with prior menstrual disorders, there were no significant changes regarding the serum levels of TG, HDL-C, TC, and LDL-C between baseline and follow-up. Moreover, no significant differences were noted in these lipid parameters at follow-up and the changes from baseline levels between two perimenopausal groups. Among postmenopausal women with prior normal menses, the serum LDL-C and TC levels decreased significantly after HRT treatment, while TG and HDL-C levels were not affected, compared with baseline. In postmenopausal women with prior menstrual disorders, no significant changes were noted in the levels of TG, HDL-C, TC, and LDL-C between baseline and follow-up. Furthermore, no significant differences in the follow-up serum lipids levels and the changes from baseline levels were demonstrated between the two postmenopausal groups.

**Table 3:** Lipid metabolism parameters in women with prior menstrual disorders and controls before and after HRT

Variable	Perimenopausal			Postmenopausal		
	Normal menstruation (*N* = 410)	Menstrual disorders (*N* = 46)	*P* value^a^	Normal menstruation (*N* = 120)	Menstrual disorders (*N* = 19)	*P* value^a^
**TG (mmol/L)**						
Before HRT	0.99 (0.76, 1.38)	0.95 (0.76, 1.33)	0.42	1.17 (0.83, 1.65)	1.19 (0.88, 1.51)	0.82
After HRT	1.08 (0.80, 1.46)	0.89 (0.66, 1.70)	0.39	1.17 (0.86, 1.57)	1.06 (0.88, 1.76)	0.66
*P* value^b^	0.001	0.08		0.44	0.93	
Δ TG^†^ (mmol/L)	0.05(−0.16, 0.33)	0.10(− 0.18, 0.50)	0.43	− 0.04(− 0.31, 0.25)	0.06(− 0.45, 0.41)	0.82
**TC (mmol/L)**						
Before HRT	5.08 (4.57, 5.64)	5.00 (4.47 5.60)	0.83	5.43 (4.71, 5.94)	5.07 (4.22, 5.43)	0.05
After HRT	4.91 (4.48, 5.53)	4.75 (4.35, 5.53)	0.47	5.24 (4.59, 5.74)	5.00 (4.56, 5.30)	0.17
*P* value^b^	0.000	0.06		0.001	0.59	
Δ TC^†^ (mmol/L)	−0.11(−0.50, 0.22)	−0.17(− 0.56, 0.22)	0.51	− 0.27(− 0.58, 0.18)	−0.05(− 0.37, 0.42)	0.19
**HDL-C (mmol/L)**						
Before HRT	1.54 (1.34, 1.79)	1.62 (1.35, 1.82)	0.38	1.47 (1.26, 1.77)	1.29 (1.19, 1.70)	0.28
After HRT	1.64 (1.39, 1.89)	1.60 (1.33, 1.85)	0.57	1.51 (1.33, 1.78)	1.36 (1.18, 1.69)	0.11
*P* value^b^	0.000	0.92		0.06	0.79	
Δ HDL-C^†^(mmol/L)	0.06(−0.07, 0.24)	−0.02(− 0.18, 0.20)	0.12	0.05(− 0.12, 0.21)	−0.01(− 0.11, 0.08)	0.30
**LDL-C (mmol/L)**						
Before HRT	2.76 (2.37, 3.25)	2.63 (2.20, 3.16)	0.27	3.07 ± 0.77	2.91 ± 0.68	0.42
After HRT	2.67 (2.21, 3.09)	2.64 (2.14, 3.12)	0.72	2.87 ± 0.74	2.79 ± 0.69	0.68
*P* value^b^	0.000	0.57		0.004	0.28	
Δ LDL-C^†^(mmol/L)	−0.11(−0.41, 0.22)	−0.19(− 0.45, 0.17)	0.98	− 0.14(− 0.63, 0.24)	− 0.18(− 0.32, 0.25)	0.82

Data are expressed as medians (25th percentile, 75th percentiles) or mean ± standard deviation as appropriate. *P* < 0.05 was considered significant. ^†^Value of the difference between the baseline and follow-up serum lipid parameters (TG, TC, HDL-C, and LDL-C). ^a^*P* values are for between-group differences by Mann-Whitney U test or independent samples t-test. ^b^*P* values are for within-group differences by Wilcoxon signed-rank test or paired-samples t test

Others: *HDL-C* high-density lipoprotein cholesterol, *HRT* hormone replacement therapy, *LDL-C* Low-density lipoprotein cholesterol, *TC* total cholesterol, *TG* triglyceride

### Glucose metabolism parameters before and after HRT therapy

No observable difference was demonstrated between women with and without a history of menstrual disorders in terms of the baseline serum levels of FPG, FIN or HOMA-IR in either the peri- or postmenopausal women (Table 4). As compared to baseline values, both peri- and postmenopausal women with prior normal menses exhibited a significant decline in FPG, FIN, and HOMA-IR after HRT treatment. In perimenopausal women with prior menstrual disorders, the serum concentrations of FIN and HOMA-IR were significantly lowered after HRT therapy, while FPG levels were not affected, compared with baseline. Serum level of FPG in postmenopausal women with prior menstrual disorders was significantly lowered following treatment, whereas the levels of HOMA-IR and FIN did not change significantly compared with baseline. Moreover, no significant difference was noticed in glucose metabolism indices at follow-up and the changes from baseline levels between participants with prior menstrual disorders and controls in either the peri- or postmenopasual women.

**Table 4:** Glucose metabolism parameters in women with prior menstrual disorders and controls before and after HRT

Variable	Perimenopausal			Postmenopausal		
	Normal menstruation (*N* = 410)	Menstrual disorders (*N* = 46)	*P* value^a^	Normal menstruation (*N* = 120)	Menstrual disorders (*N* = 19)	*P* value^a^
**FPG (mmol/L)**						
Before HRT	5.23 (4.93, 5.53)	5.14 (4.85, 5.49)	0.25	5.33 (5.00, 5.63)	5.50 (4.84, 5.97)	0.40
After HRT	5.13 (4.87, 5.41)	5.02 (4.81, 5.58)	0.49	5.12 (4.93, 5.41)	5.18 (4.85, 5.32)	0.75
*P* value^b^	0.000	0.64		0.000	0.002	
Δ FPG^†^ (mmol/L)	−0.10(− 0.42, 0.18)	0.01(− 0.37,0.19)	0.36	− 0.17(− 0.44, 0.07)	−0.37(− 0.67, 0.06)	0.11
**FIN (mU/L)**						
Before HRT	5.60 (4.30, 7.34)	5.82 (4.73,7.63)	0.26	5.83 (4.10, 8.16)	6.25 (3.40, 7.73)	0.93
After HRT	5.03 (3.79, 6.37)	5.10 (4.08, 7.03)	0.36	4.75 (3.50, 6.20)	4.50 (3.10, 6.10)	0.97
*P* value^b^	0.000	0.006		0.000	0.12	
Δ FIN^†^ (mU/L)	−0.70(−2.34, 0.60)	−0.60(−1.91, 0.00)	0.86	−1.07(−2.51, 0.36)	− 0.20(−3.10, 1.10)	0.80
**HOMA-IR**						
Before HRT	1.29 (0.98, 1.74)	1.37 (1.05,1.83)	0.38	1.37 (0.96, 1.96)	1.62 (0.84, 1.88)	0.81
After HRT	1.15 (0.85, 1.51)	1.20 (0.88, 1.61)	0.43	1.05 (0.76, 1.47)	1.06 (0.70, 1.47)	1.00
*P* value^b^	0.000	0.008		0.000	0.05	
Δ HOMA-IR^†^	−0.17(−0.59, 0.13)	−0.18(− 0.49, 0.09)	0.89	− 0.28(− 0.67, 0.03)	−0.19(− 0.92, 0.15)	0.88

Data are expressed as medians (25th percentile, 75th percentiles). *P* < 0.05 was considered significant. ^†^Value of the difference between the baseline and follow-up serum glucose metabolism parameters (FPG, FIN, and HOMA-IR). ^a^*P* values are for between-group differences by Mann-Whitney U test or independent samples t-test. ^b^*P* values are for within-group differences by Wilcoxon signed-rank test or paired-samples t test

Others: *FIN* fasting insulin, *FPG* fasting plasma glucose, *HOMA-IR* homeostasis model assessment of insulin resistance, *HRT* hormone replacement therapy

## Discussion

This retrospective multicenter analysis is the first one of which we are aware to compare the effects of HRT use on glucose and lipid metabolism in peri- and postmenopausal women with prior menstrual disorders and women with prior normal menses. Our findings demonstrated that HRT showed more obvious within-group improvements in women with prior normal menses in terms of glucose and lipid metabolism parameters during perimenopause and after menopause, but there is no significant between-group difference.

As is well-known, PCOS is the most frequent explanation for irregular menses [6, 7]. Women with a history of irregular cycles had higher coronary heart disease mortality, which they attributed to a high proportion of PCOS and its related metabolic disorders among women with menstrual irregularity [28, 29]. However, some previous studies have suggested that identifying postmenopausal women with PCOS is difficult because of their amelioration of the classic phenotype of the syndrome with aging [12, 13]. Evidence indicated that the hyperandrogenism and insulin resistance phenotype of women with PCOS partly improved with aging [12, 30]. According to Talbott et al. [31], no difference was observed in LDL-C and TC levels between older PCOS cases (≥ 40 years) and their age-matched controls. In addition, Cibula et al. [32] showed that there was no difference in BMI, waist circumference, waist-hip ratio, mean concentrations of lipids or fasting glucose between perimenopausal women with a history of PCOS and controls. Similarly, our results showed that there was no significant differences in any of the glucose and lipid metabolism indicators at baseline between women with and without prior menstrual disorders in either the peri- or postmenopausal women, suggesting that the PCOS phenotype improve with aging.

Due to the decline in estrogen, menopause is accompanied by atherogenic dyslipidemia, characterized by elevated serum levels of LDL-C and TG and reduced HDL-C levels [16]. Up to now, the effects of exogenous estrogen on serum lipid profiles in postmenopausal women have been extensively explored. Consistent results from several large randomized controlled trials indicated that HRT produced significant increases in HDL-C levels, and reductions in LDL-C levels [20–23]. Generally, estrogen regulates gene expression involved in lipid metabolism by signaling through estrogen receptors. Evidence indicates that estrogens decrease the activity of hepatic lipase and increase the formation of HDL and HDL-lipoprotein (apo AI) in the liver, which may account for the associated increase in HDL. Meanwhile, estrogen increases the clearance of LDL from the circulation by the upregulation of the LDL receptor. Furthermore, estrogen lowers TC concentrations primarily from a decrease in LDL levels. The increase in TG induced by oral estrogens is a consequence largely of increased hepatic secretion of very low density lipoprotein [33–35]. Overall, estrogen has a certain protective effect on lipid metabolism. Nonetheless, little attention has been paid to whether HRT use can also exhibit the favorable effects on metabolic homeostasis in peri- and postmenopausal women with a history of menstrual disorders. Hence, in this paper, the effects of HRT on glucose and lipid metabolism were compared in peri- and postmenopausal women with prior menstrual disorders and controls. Our analyses showed that HRT led to a marked decline in TC and LDL-C levels in both peri- and postmenopausal women with prior normal menses, increased TG and HDL-C levels in perimenopausal controls but did not affect TG and HDL-C levels in postmenopausal controls respectively, compared with baseline. However, there were no significant changes regarding the levels of TG, HDL-C, TC, and LDL-C after HRT in either the peri- or postmenopausal women with prior menstrual disorders, compared with baseline. In addition, no significant differences were observed in any of the lipid metabolism indicators at follow-up, as well as the changes from baseline levels between women with and without prior menstrual disorders in either the peri- or postmenopausal women.

Similarly, the positive effect of endogenous estrogen on glucose homeostasis was lost after menopause [36]. Many previous large-scale randomized controlled trials have verified that HRT led to a decline in fasting glucose levels and decreased the incidence of type 2 diabetes [37–40]. The beneficial effect of estrogen therapy on glucose homeostasis may be the result of modulation hepatic insulin sensitivity via estrogen receptor-α [41]. However, no study of which we are aware has been conducted to investigate whether HRT show the similar favourable effects on glucose metabolism indexes in peri- and postmenopausal women with a history of menstrual disorders. In this study, we found that HRT significantly decreased FIN and HOMA-IR in perimenopausal users, as well as FPG level in postmenopausal users with prior menstrual disorders, compared with baseline. In addition, HRT resulted in improvements of FPG, FIN, and HOMA-IR levels in both peri- and postmenopausal women with prior normal menses, compared with baseline. However, there were no significant differences in FPG, FIN, or HOMA-IR levels at follow-up, as well as the changes from baseline levels between women with prior menstrual disorders and controls in either the peri- or postmenopausal women.

Based on the present results, women with prior normal menses showed more obvious within-group improvements in glucose and lipid metabolism after HRT. However, there was no significant between-group difference in peri- and postmenopausal women with and without a history of menstrual disorders at any of the two test occasions in any of the glucose and lipid metabolism parameters. The nonsignificant between-group difference could have several reasons. One is that the classic phenotype of PCOS may improve with aging [12, 30–32]. Another explanation is that the diagnosis of PCOS in menopausal women with prior menstrual disorders could not be confirmed due to lack of complete information on clinical or biochemical hyperandrogenism among the participants. In addition, the small sample size of women with prior menstrual disorders might explain these findings to some extent. Hence, additional studies based upon a larger sample size and a well-defined PCOS population in the peri- and postmenopausal years are required for confirmation.

There are several potential limitations to this study. First, our investigation was a retrospective analysis. Second, the sample size of women with prior menstrual disorders was too small. Third, selection and recall bias existed in the recruitment of the participants. Given these limitations, further prospective studies with larger sample sizes, homogeneous hormone therapies (type, dose of estrogen and progestogen) and longer follow-up duration are required.

## Conclusion

In conclusion, our present results indicate that HRT shows more obvious within-group improvements in glucose and lipid metabolism in peri- and postmenopausal women with prior normal menstruation, but no statistically significant difference was noted in the between-group analysis. Further prospective studies are required for confirmation.

## Data Availability

The datasets used and/or analysed during the current study are available from the corresponding author on reasonable request.

## Abbreviations

BMI: Body mass index
CVD: Cardiovascular disease
E2: Estradiol
FIN: Fasting insulin
FPG: Fasting plasma glucose
FSH: Follicle-stimulating hormone
HDL-C: High-density lipoprotein cholesterol
HOMA-IR: Homeostasis model assessment of insulin resistance
HRT: Hormone replacement therapy
LDL-C: Low-density lipoprotein cholesterol
LH: Luteinizing hormone
PCOS: Polycystic ovary syndrome
SD: Standard deviation
TC: Total cholesterol
TG: Triglycerides
